# The Decline and Fall (?) of Tuberculosis.—I

**Published:** 1922-04

**Authors:** Edgar L. Collis

**Affiliations:** (Mansel Talbot Professor of Preventive Medicine, Welsh National School of Medicine)


### THE DECLINE AND FALL (?) OF TUBERCULOSIS.

By EDGAR L. COLLIS, M.A., M.D., M.R.C.P. (Mansel Talbot Professor of Preventive Medicine, Welsh National School of Medicine.)

### Introduction.

A CAMPAIGN supported by public funds is being waged against tuberculosis. Two main lines of attack are being pursued; one aims at curing those already affected, the other at preventing the occurrence of new cases. The object of this article is to deal with the latter. A review of how tuberculosis occurs in the community; of how other infectious diseases have been brought under control and of the means which have brought them under control; of how the incidence of tuberculosis has behaved in the past; and of where, if anywhere, signs of weakness can be detected, may suggest how and where the attack should be pressed.

### The Occurrence of Tuberculosis.

History tells that for long ages tuberculosis, at any rate in its pulmonary form, has been endemic among civilised nations; there is no evidence of its occurring in epidemics. Nevertheless, the disease, since the work of Villemin in the middle of last century and the discovery by Koch of the tubercle bacillus, has been recognised to be infectious. Clinically the disease exhibits no definite incubation period and runs no definite course; herein it differs notably from the acute epidemic diseases. It passes from one host to another either by ingestion or by inhalation.

### Other Infectious Diseases.

Some diseases recognised to be infectious have been brought more or less completely under control. Malaria, which used to be endemic in this country, with seasonal epidemics now recognised to have depended upon the breeding of mosquitoes[1], has been banished by draining the marsh lands, and so greatly reducing the number of carrier-mosquitoes. Typhus or gaol fever, now known to be transmitted by the louse, gave way as personal hygiene was rendered possible by the introduction of plentiful pure water supply; and, in the case of armies, before prophylactic inoculation. Scarlet fever is often claimed to have diminished owing to isolation, but the suggestion recently put forward by Hamer[2] that the unknown infecting agent is transmitted by the common flea (pulex irritans) may group this disease with typhus, by attributing diminution of the disease, not to isolation, but to personal hygiene; and may explain its epidemiology by the presence or absence of an unusual number of fleas. The pneumonia group, although less pronouncedly infectious, may also be instanced on account of the control established over it on the Rand in South Africa by prophylactic inoculation of native labour[3]. Inoculation has not only reduced the incidence of the disease among the natives, but has been accompanied by a fall in general mortality over and above what is due to the reduction in pneumonia deaths. This result is an interesting one, and is probably due to the lessened case incidence of pneumonia of which the reduced mortality is an indication. Each non-fatal case represents a member of the community damaged, and more likely to fall a prey to other forms of disease. Precisely the same sequence of events was noted in Kuala Lumpur between 1911 and 1919, when, by an anti-mosquito campaign, the malaria death-rate was lowered from 9·87 to 4·69; the general death-rate during this period fell from 39·02 to 26·36[1].

Diphtheria presents an interesting example of a disease concerning which the infecting agent, a definite antidote, and a prophylactic serum, have all been known for twenty-five years; a disease which, like scarlet fever, has been dealt with by isolation, but, notwithstanding, a disease the case incidence of which in the last fifty years has shown no marked diminution (although the case mortality has been checked by the use of antitoxin). Other infectious diseases such as measles, mumps, chicken pox, and whooping cough, all of which recur in epidemics, continue practically unchecked (*see* Table I.).

TABLE I.—ANNUAL (STANDARDISED) MORTALITY FROM SEVERAL CAUSES PER MILLION LIVING AT ALL AGES.*

| Cause of Death. | 1861–70 | 1871–80 | 1881–90 | 1891–1900 | 1901–10 |
|---|---|---|---|---|---|
| Small Pox | 149 | 228 | 45 | 14 | 13 |
| Enteric | — | 321 | 199 | 175 | 91 |
| Scarlet Fever | 857 | 625 | 300 | 152 | 109 |
| Pneumonia | 990 | 937 | 1034 | 1214 | 1254 |
| Diphtheria | 166 | 108 | 148 | 254 | 183 |
| Measles | 377 | 322 | 392 | 399 | 318 |
| Whooping Cough | 450 | 434 | 399 | 364 | 286 |
| Phthisis | 2590 | 2231 | 1810 | 1418 | 1143 |

* Supplement to 75th Annual Report of Registrar-General, Part III, 1919. (Cd. 8002.)

supplies of water. Small-pox surrendered before vaccination and is held at bay by the same weapon combined with rigid isolation. The Typhoid group has given way before uncontaminated food, and a

All these diseases have been or are endemic in the country, with periods of epidemic spread. Moreover, their prevalence tends to vary with the housing conditions of the population, one-room dwellings having a higher incidence than two rooms, and two than three[4]. Herein these diseases resemble tuberculosis; but they differ from it in that the death-rates they cause rise or fall independently of the general death-rate; while the death-rate from tuberculosis is highly correlated with the general death-rate (*see* Table II.). The reason for this possibly is that, while the incidence of these diseases is affected, as is that of tuberculosis, by those social conditions which are held to lower resistance, yet their prevalence is more influenced by their epidemic occurrence, the underlying causes of which, *e.g.*, an increase in insect carriers, are not determined by the more constant influences which determine the general death-rate.

Table II.—Comparative Mortality of England and Wales Since 1851.*

| Period. | Males. | | Females. | |
|---|---|---|---|---|
| | Phthisis. | All Causes. | Phthisis. | All Causes. |
| 1851–60...... | 100 | 100 | 106 | 91 |
| 1861–70...... | 97 | 102 | 96 | 91 |
| 1871–80...... | 88 | 99 | 79 | 85 |
| 1881–90...... | 73 | 90 | 62 | 78 |
| 1891–1900... | 61 | 88 | 46 | 75 |
| 1901–10...... | 50 | 75 | 35 | 62 |

* The death rates are expressed as percentages of the male rate for 1851–60, which was for All Causes 22·1 per 1,000, and for Phthisis 2·6 per 1,000.

**Effect of Isolation.**

If the doubtful case of scarlet fever be disregarded there is no clear evidence that isolation alone has profoundly influenced the prevalence of any of these infectious fevers. On the contrary, isolated diphtheria and non-isolated measles have, so far as case incidence is concerned, behaved in much the same way in the last forty years (*see* Table I). Evidence rather points to success being associated only with interference with the transmitting agent of infection, and to the epidemic character of those diseases in which means of transmission is recognised, depending upon the life-history of the mosquito, the louse and the flea, rather than upon any increased virulence in the infective germ, or any decreased resistance in the community.

The possibility that isolation has had but little effect upon these infectious fevers does not rule out the possibility of its proving valuable in the case of tuberculosis, which differs clinically and epidemiologically from these fevers. Certainly there is no positive evidence in favour of isolation for tuberculosis; but in the case of a more closely allied disease, leprosy, there is some evidence that isolation has value.

In Norway as long ago as 1861 the principle of segregating cases of leprosy was adopted, but at first only 27 per cent. of the known cases were segregated. Yet the disease commenced to decline, and was practically under control by 1901-5, although even then only 52 per cent. of the cases were segregated[5]. The possibility must, however, be faced that the decline may have been due to such influences as have led to the decline of tuberculosis in this country and not to segregation.

**Epidemiology and Immunity.**

A point of interest is that epidemics of these acute fevers tend to last a definite period; thus an epidemic of scarlet fever or measles usually lasts about four months, of smallpox, malaria or plague about three months, and of influenza six weeks. This case incidence among the population resembles the manifestations of the disease in individuals which have definite periods of incubation and, for uncomplicated cases, run definite and regular courses. Here there would appear to be a definite rule that all fevers that run a definite clinical course, and are infectious, attack the community in epidemics which last for definite periods.

The fact that an attack, unless fatal, of any one of these infectious fevers, terminates in a definite period and does not continue indefinitely, is held to be due to the body establishing, through the creation of antibodies, an antidote to the poison of the infecting germ, an antidote which persists for a variable period after the disease is recovered from, and confers for as long as it lasts an immunity to subsequent attack of the same germ. Evidence from these diseases suggests that in the case of any disease an attack of which does not run a definite course and then terminate, the body does not react by creating a sufficiency of antibodies to neutralise the poison of the invading germ and to confer protection against subsequent attack. And the non-occurrence of such a disease in epidemics would tend to confirm this suggestion.

Tuberculosis is such a disease; it has no definite incubation period and runs no definite and regular clinical course. Further, this indefiniteness is reflected in the way in which the community is attacked, since the disease is endemic but shows little or no tendency to occur in epidemics. There is then (arguing on the somewhat dangerous basis of analogy) but little probability that in the case of tuberculosis the body reacts by preparing a useful supply of antibody capable of terminating an attack, or of protecting the individual against further attack. This conclusion is important in view of the attention directed at present to obtaining through serology a means for controlling tuberculosis, largely based on the contention that white races among whom the disease is endemic are at birth virgin soil for attack, but that when compared with native races among whom the disease is not endemic they exhibit in adult life some degree of immunity to the disease which is held to be acquired through reacting to infection during youth. Before, however, this view is accepted two explanations should be considered of the fact that white races, when exposed to the same risk of infection as native races, among whom the disease is absent normally, certainly do not succumb to the disease as do the natives. On the one hand whites are the descendants of many generations from among whom the disease, being endemic, has been steadily removing the more susceptible, so that the present generation descended from the less susceptible has come, by a process of the survival of the fittest, to possess a certain resisting power—quite a different thing from an acquired immunity; on the other hand, natives when brought into contact with civilisation are exposed to many new influences, dietetic and environmental, which may lower any natural resistance they may possess to tuberculosis.

Evidence drawn from the United States is favourable to this view. In that country the white population represents the descendants of a race among whom the disease has for long been endemic and from among whom it has, according to the view put forward, been removing in each generation the more susceptible; *i.e.*, the white population possesses a certain amount of inherited resistance. The coloured population represents the descendants of a race among whom the disease has only been endemic since the race came in close contact with whites; it should, therefore, possess less inherited resistance. Exposure to infection in a town like New York, since the infection is held to be ubiquitous among the white population, must be considered to be approximately equal for both white and coloured. Each member of the coloured population should then have the same chance as a white person of establishing during his own lifetime immunity against the disease; and on the theory that an attack of the disease confers a degree of immunity, of such potency as to influence mortality statistics, the mortality among the coloured population should approximate to that among the whites. On the other hand, the theory that the whites have inherited a resistance creates an expectation that the whites would suffer less and that the coloured population with less inherited resistance would suffer more. Moreover, any influence liverse to natural resistance, such as food restriction which occurred during the war, might be expected to have more effect on the less resistant coloured than on the more resistant white population. What effect upon the two classes should be expected from a subsequent increase in food supply is hard to foretell.

Reference to the data of Table III. shows that the death-rate from tuberculosis among the coloured population is far higher than among the white; it is highest in Kentucky where the white rate is highest; it is lowest in South Carolina where the white rate is lowest. During the war period a rise took place for both groups of the population which by 1918, taking the 1916 death-rate as a standard, amounted in New York for whites to 4·1 per cent. and for coloured to 12·5 per cent.; in Pennsylvania the rise was 13·1 per cent. for whites, and 19·7 per cent. for the coloured. In the warm Southern States, where food is normally plentiful and the energy requirements are lower, the war period does not appear to have adversely affected the normally low death rates.

After the war, when there was a boom in trade and food became proportionately plentiful, a fall in the tuberculosis rate took place which by 1920, compared with the 1916 standard, reached in New York for whites 25·5 per cent. and for coloured 14·1 per cent.; in Pennsylvania it reached 22·2 per cent. for whites and 13·2 per cent. for coloured. In the South States the fall affected the two groups differently; it amounted by 1920 in North Carolina to 18·8 per cent. for the whites and 22·1 per cent. for coloured, and in South Carolina to 2·2 per cent. for whites, and 21·7 per cent. for coloured.

These statistics may fairly be held not to support the theory of immunity, but to support that of inherited resistance.

A community in which an infectious disease has been long endemic might come to possess a power for repelling the disease in one or two ways:—

Either the disease might attack the whole community when, if it conferred immunity on the survivors, it would lead to its own eradication, unless a new generation were constantly springing up to carry on the infection. An endemic disease behaving in this way might be expected to attack principally the young non-immunised portion of the community and to manifest itself in epidemics, the occurrence of which would be determined by the length of time needed for a new non-immunised generation to come into existence and also by any factor concerned with transmission of the infection (such as seasonal prevalence of insect carriers). The specific fevers of juvenile life may be quoted as possible instances of this type.

Or if the disease conferred no such immunity on the survivors, it might, by killing off over long periods

Table III.—Death Rates Among White and Coloured Populations from Tuberculosis (all forms) in Certain Areas of the United States, 1916–1920.

| Area. | Race. | Rate per 100,000 population. | | | | |
|---|---|---|---|---|---|---|
| | | 1916 | 1917 | 1918 | 1919 | 1920 |
| Registration Area | Mixed | 142·1 | 147·1 | 150·0 | 125·6 | 114·2 |
| Kentucky | White | 163·7 | 166·1 | 169·3 | 143·1 | 130·3 |
| | Coloured | 467·4 | 486·1 | 466·0 | 367·8 | 363·8 |
| New York | White | 155·4 | 161·1 | 161·8 | 134·4 | 115·8 |
| | Coloured | 422·2 | 429·4 | 475·0 | 455·7 | 362·0 |
| Maryland | White | 148·6 | 150·8 | 163·1 | 129·4 | 110·4 |
| | Coloured | 420·8 | 413·6 | 423·4 | 344·2 | 326·6 |
| Pennsylvania | White | 125·4 | 129·1 | 141·8 | 111·4 | 97·5 |
| | Coloured | 373·5 | 438·0 | 447·0 | 351·3 | 324·1 |
| Tennessee | White | — | 155·6 | 151·5 | 126·6 | 129·3 |
| | Coloured | — | 374·5 | 432·5 | 344·9 | 312·6 |
| Virginia | White | 118·4 | 109·2 | 126·1 | 97·5 | 94·8 |
| | Coloured | 301·7 | 307·6 | 315·9 | 272·0 | 256·2 |
| North Carolina | White | 101·5 | 94·2 | 94·7 | 86·0 | 82·4 |
| | Coloured | 249·4 | 236·4 | 238·8 | 205·9 | 194·2 |
| South Carolina | White | 67·0 | 70·0 | 67·9 | 68·0 | 65·5 |
| | Coloured | 217·0 | 213·0 | 215·4 | 199·0 | 172·0 |

of time those least able to resist it, bring into existence, by a species of natural selection, a community more able to resist it, not because the community possessed a specific immunity conferred by specific anti-bodies, but because it inherited that power of resistance which enabled previous generations to resist or to throw off the disease. The disease in this latter case would immediately attack any newcomers into the world who failed to inherit the power held not to confer upon those who recover any specific immunity such as is conferred by the acute specific fevers. The prevalence of tuberculosis at different periods of life is suggestive that it conforms to this type (*see* Table IV.).

Deduction.—Tuberculosis is a disease of which the clinical manifestations and epidemiology do not suggest that it establishes against itself an immunity comparable with that established by other infectious

Table IV.—Mortality per Million Living from Tuberculosis (All Forms) at Various Age Periods.

| Sex. | Years. | Age Period. | | | | | | | | | | |
|---|---|---|---|---|---|---|---|---|---|---|---|---|
| | | 0– | 5– | 10– | 15– | 20– | 25– | 35– | 45– | 55– | 65– | 75 & over |
| Males | 1851–60 | 6323 | 1225 | 1102 | 2636 | 4245 | 4163 | 4119 | 3957 | 3479 | 2573 | 1061 |
| | 1901–10 | 3129 | 636 | 463 | 997 | 1744 | 2158 | 2622 | 2934 | 2574 | 1686 | 668 |
| Females | 1851–60 | 5232 | 1201 | 1595 | 3731 | 4430 | 4690 | 4293 | 3236 | 2523 | 1783 | 834 |
| | 1901–10 | 2636 | 698 | 710 | 1250 | 1425 | 1651 | 1710 | 1449 | 1186 | 894 | 494 |

of resistance, and subsequently, as life continued, would claim its victims among those whose power of resistance was at any time lowered. In such a case the disease might be expected to claim a crop of victims soon after birth, and then abate in prevalence, and to increase in prevalence again as the strain and stress of life with advancing years lowered vitality. The incidence of the disease might be expected in such a case to maintain this age distribution whether its incidence rose or fell. Reversing the argument, an infectious disease which exhibits such a prevalence according to the age periods of its victims may be diseases. Further, its prevalence by age periods is in conformity with this suggestion.

References.

(1) Watson, Malcolm. "Prevention of Malaria in the Malay States." Second edition, 1921. John Murray.

(2) Annual Report of the London County Council for 1920, dealing with Public Health, p. 86.

(3) Statement of Evidence submitted by the South African Institute of Medical Research to the Low Grade Mines Commission. 1919. Johannesburg.

(4) Kinloch, J. P. "Metabolism and Disease." Proc. Roy. Soc. Medic., Jan., 1922.

(5) Newsholme, A. "The Prevention of Tuberculosis." Methuen & Co., 1908.

(*To be continued.*)

### PELLAGRA IN SCOTLAND.

At the Royal Edinburgh Hospital last year three female patients fell victims to pellagra, a very common disease in Italy and sunnier climes than ours. A fourth, who also suffered, has apparently made a good recovery. In all these cases the diagnosis was confirmed by Professor Francis Darby Boyd, who, during the war, had great experience of this disease in Palestine and Egypt. Pellagra was not known to exist in Great Britain till a Shetland girl was discovered suffering from it in the West House in the year 1911, whose case was described by Dr. Dods Brown and Dr. Cranston Low. Attention having thus been drawn to the subject, a considerable number of other cases were found scattered all over the country. Dr. Watson, pathologist to the Rainhill Asylum, containing over 2,000 patients, found, during the years 1913 to 1918, no less than 36 cases, of which all died with the exception of six.

It was believed at one time that diseased maize was the cause of pellagra among an ill-fed population, but these cases had all developed, previous to admission, among the well-to-do, and none of them appear ever to have tasted maize. Dr. Dudgeon, of the Kanka Asylum, Egypt, has suggested that as belladonna poisoning may occur from eating rabbits that have fed on belladonna leaves, so these patients may have become affected by eating chickens fed on diseased maize, but even this possibility can be excluded in some of the cases. Professor Robertson is of opinion that the disease, which affects the brain and nervous system, may have been caused in cases by some form of food deficiency, not because the food itself was not provided, but because, owing to digestive and intestinal disorders which are almost invariably present, some element essential for the health of the nervous system was not being digested and absorbed.

The disease is definitely diagnosed by a characteristic eruption, resembling sunburn, which appears on the hands and face, usually after exposure to sunlight. As the Morningside patients live so much in verandahs in the open air, these symptoms have an exceptionally favourable opportunity given them of manifesting themselves and rendering diagnosis easy, especially in a hot summer. The question may well be asked, how many unknown cases of pellagra exist in asylums and among the general population with nervous and mental symptoms, in which the disease has not been diagnosed because the characteristic eruption has never manifested itself. Professor Robertson believes that its apparent infrequency may be partly due to the lesser degree of sunshine which we enjoy in this misty northern country.

